# EPR pairing dynamics in Hubbard model with resonant *U*

**DOI:** 10.1038/srep18323

**Published:** 2016-01-05

**Authors:** X. Z. Zhang, Z. Song

**Affiliations:** 1 School of Physics, Nankai University, Tianjin 300071, China; 2 College of Physics and Materials Science, Tianjin Normal University, Tianjin 300387, China

## Abstract

We study the dynamics of the collision between two fermions in Hubbard model with on-site interaction strength *U*. The exact solution shows that the scattering matrix for two-wavepacket collision is separable into two independent parts, operating on spatial and spin degrees of freedom, respectively. The S-matrix for spin configuration is equivalent to that of Heisenberg-type pulsed interaction with the strength depending on *U* and relative group velocity *v*_*r*_. This can be applied to create distant EPR pair, through a collision process for two fermions with opposite spins in the case of |*v*_*r*_/*U*| = 1, without the need for temporal control and measurement process. Multiple collision process for many particles is also discussed.

Pairing is the origin of many fascinating phenomena in nature, ranging from superconductivity to quantum teleportation. Owing to the rapid advance of experimental techniques, it has been possible both to produce Cooper pairs of fermionic atoms and to observe the crossover between a Bose-Einstein condensate and a Bardeen-Cooper-Schrieffer superfluid^1^,^2^,^3^. The dynamic process of pair formation is of interest in both condensed matter physics and quantum information science. On one hand, the collective behavior of pairs gives rise to macroscopic properties in many-body physics. On the other hand, a single entangled pair is a promising quantum information resource for future quantum computation.

In recent years, the controlled setting of ultracold fermionic atoms in optical lattices is regarded as a promising route to enabled quantitative experimental tests of theories of strongly interacting fermions^4^,^5^,^6^,^7^. In particular, fermions trapped in optical lattices can directly simulate the physics of electrons in a crystalline solid, shedding light on novel physical phenomena in materials with strong electron correlations^4^,^8^,^9^. A major effort is devoted to simulate the Fermi-Hubbard model by using ultracold neutral atoms^10^,^11^,^12^. This approach offers experimental access to a clean and highly flexible Fermi-Hubbard model with a unique set of observables^13^ and therefore, motivate a large number of works on Mott insulator phase^14^,^15^ and transport properties^16^,^17^, stimulating further theoretical and experimental investigations on the dynamics of strongly interacting particles for the Fermi Hubbard model.

In this paper, we study the dynamics of the collision between two fermions with various spin configurations. The particle-particle interaction is described by Hubbard model, which operates spatial and spin degrees of freedom in a mixed manner. Based on the Bethe ansatz solution, the time evolution of two fermonic wave packets with identical size is analytically obtained. We find that the scattering matrix of the collision is separable into two independent parts, operating on spatial and spin degrees of freedom, respectively. The scattered two particles exhibit dual features. The spatial part behaves as classical particles, swapping the momenta, while the spin part obeys the isotropic Heisenberg-type exchange coupling. The coupling strength depends on the Hubbard on-site interaction and relative group velocity of two wavepackets. This finding can be applied to create distant EPR pair, through a collision process for two fermions with opposite spins without the need for temporal control and measurement process. Multiple collision process for many particles is also discussed.

## Results

### Fermi-Hubbard Model

A one-dimensional Hubbard Hamiltonian on an *N*-site ring reads


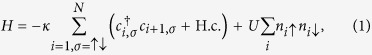


where 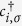 is the creation operator of the fermion at the site *i* with spin 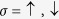 and *U* is the on-site interaction. The tunneling strength and the on-site interaction between fermions are denoted by 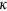 and *U*. For the sake of clarity and simplicity, we only consider odd-site system with 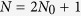, and periodic boundary condition 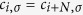.

Now based on the symmetry analysis of the Hamiltonian (1) as shown in Methods section, we can construct the basis of the two-fermion invariant subspace as following


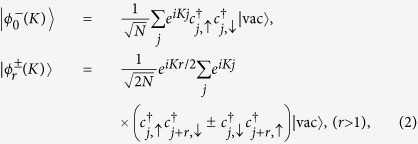


and


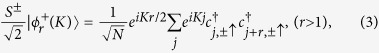


where *K* is the momentum vector, indexing the subspace. These bases are eigenvectors of the operators 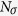, 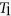, 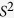 and 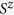. The corresponding definitions of the operators are detailed in Methods section. Straightforward algebra yields


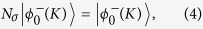



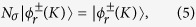



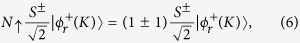


and


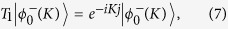



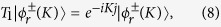



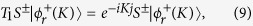


while


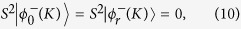



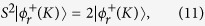



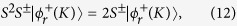


and


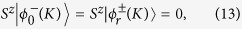



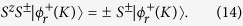


Then there are four invariant subspaces with 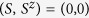, 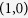, and 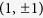 involved.

### Dynamics of wavepacket collision

We now want to investigate the dynamics of two-wavepackets collision based on the two-particle solution, which is shown in Methods section. We begin with our investigation from the time evolution of an initial state


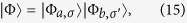


which represents two separable fermions *a* and *b*, with spin *σ* and 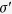, respectively. Here


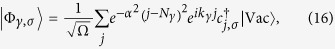


with 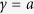, *b* and 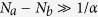, is a wavepacket with a width 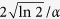, a central position 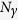 and a group velocity 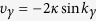. We focus on the case 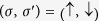. The obtained result can be extended to other cases. In order to calculate the time evolution of state 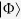, two steps are necessary. At first, the projection of 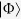 on the basis sets 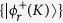 and 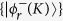 can be given by the decomposition.


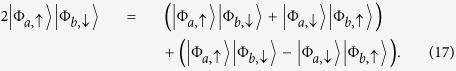


Secondly, introducing the transformation.


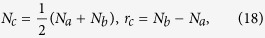



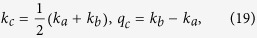



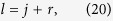


and using the identities


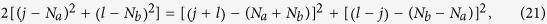



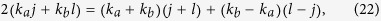


we have


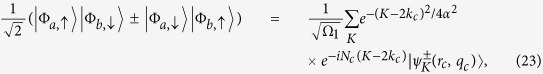


with


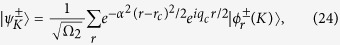


where 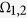 is the normalized factor.

We note that the component of state 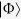 on each invariant subspace indexed by *K* represents an incident wavepacket along the chain described by 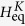 that is presented in Eq. (59) of Methods. This wavepacket has width 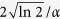, central position 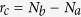 and group velocity 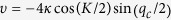. Accordingly, the time evolution of state 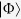 can be derived by the dynamics of each sub wavepacket in each chain 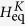, which eventually can be obtained from Eq. (60) of Methods section. According to the solution, the evolved state of 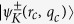 can be expressed approximately in the form of 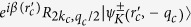, which represents a reflected wavepacket. Here 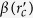 is an overall phase, as a function of 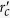, the position of the reflected wavepacket, being independent of *U* and the 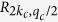 is the reflection amplitude demonstrated in Eq. (61) of Methods. In addition, it is easy to check out that, in the case with 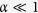, the initial state distribute mainly in the invariant subspace 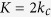, where the wavepacket moves with the group velocity 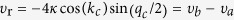. Then the state after collision has the approximate form


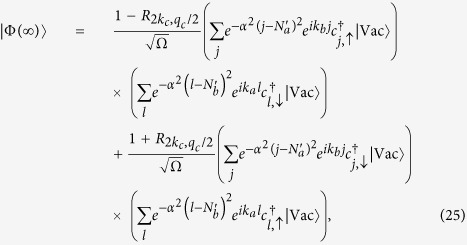


which also represents two separable wavepackets at 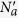 and 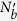 respectively. Here Ω is the normalized factor and an overall phase is neglected. We would like to point out that the key point to achieve this interesting phenomenon is that the coordinates of the two particles can be decomposed into two independent parts in term of the center of mass coordinates and relative coordinate. We do not preclude that the initial two-particle state possessing the different shape may get to the same conclusion. However, the strict proof of this general condition is hardly obtained. For the sake of simplicity and clarity, we confine our disscussion to the case of two wavepackets 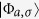 and 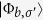 with same shape.

### Equivalent Heisenberg coupling

Now we try to express the two-fermion collision in a more compact form. We will employ an S-matrix to relate the asymptotic spin states of the incoming to outcoming particles. We denote an incident single-particle wavepacket as the form of 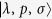, where 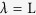, R indicates the particle in the left and right of the collision zone, *p* the momentum, and 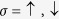 the spin degree of freedom. In this context, we give the asymptotic expression for the collision process as


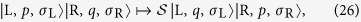


where the S-matrix


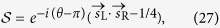


governs the spin part of the wave function. Here 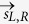 denotes spin operator for the spins of particles at left or right, 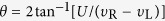, where 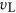 and 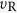 represent the group velocity of the left and right wavepacket, respectively. Together with the scattering matrix 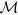 for spatial degree of freedom


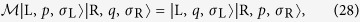


we have a compact expression


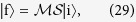


to connect the initial and final states. In general the total scattering matrix has the form of exp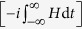, which is not separable into spatial and spin parts. Then Eq. (29) is only available for some specific initial states, e.g., spatially separable two-particle wavepackets with identical size. This may lead to some interesting phenomena.

It is interesting to note that the scattering matrix for spin is equivalent to the propagator


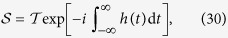


for a pulsed Heisenberg model with Hamiltonian


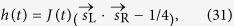


with 
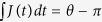
. Here 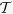 is time-ordered operator, which can be ignored since only the coupling strength 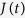 is time dependent. This observation accords with the fact that, in the large positive *U* case, the Hubbard model scales on the 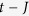 model^18^,^19^, which also includes the NN interaction term of isotropic Heisenberg type. Recently, we note that several perturbative techniques have been proposed to construct an effective spin Hamiltonian in the context of the one-dimensional interacting confined system^20^,^21^,^22^,^23^,^24^. Nevertheless, they confine their results to the strongly interaction regime. Here, we need to stress that the derivation of the Eq. (31) is obtained from the dynamic aspect and does not dependent on the approximation of large interaction strength *U*, which is different from that of those works.

The aforementioned scattering matrix for spin also indicates that the effect of collision on two spins is equivalent to that of time evolution operation under the Hamiltonian 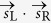 at an appropriate instant. In this sense, the collision process can be utilized to implement two-qubit gate. For two coupled-qubit system, the time evolution operator is simply given by


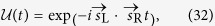


yielding


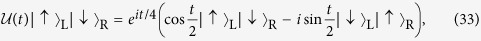


where 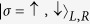 denotes qubit state. We can see that at instants 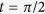 and *π*, the evolved states become


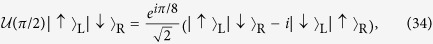



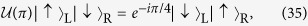


which indicates that 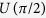 and 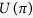 are entangling and swap operators, respectively. In practice, such protocols require exact time control of the operation.

Comparing operator 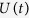 and the S-matrix in Eq. (27), we find that two-qubit operations can be performed by the collision process, where *U* and relative group velocity 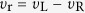 are connected to the evolution time by the relation


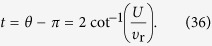


**Figure 1:**
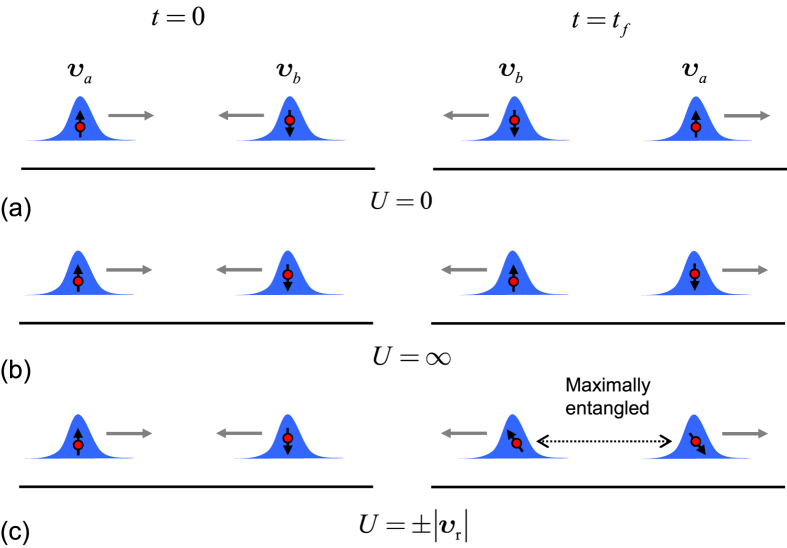
Schematic illustration of the collision process of two separated fermionic wavepackets with opposite spin orientations for three typical values of *U*. In all cases, the collisions result in momentum swap, but different spin configurations: (**a**) 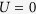, swap; (**b**) 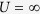, unchange; (**c**) 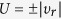, maximal entanglement.

Then we can implement entangling and swap gates for two flying qubits via dynamic process. To demonstrate the result, we consider several typical cases with 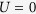, 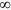, and 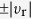, which correspond to the operations of swap, standby, and entanglement, respectively. The collision processes are illustrated schematically in Fig. 1. The advantage of such a scheme is that the temporal control is replaced by pre-engineered on-state interaction *U*.

**Figure 2:**
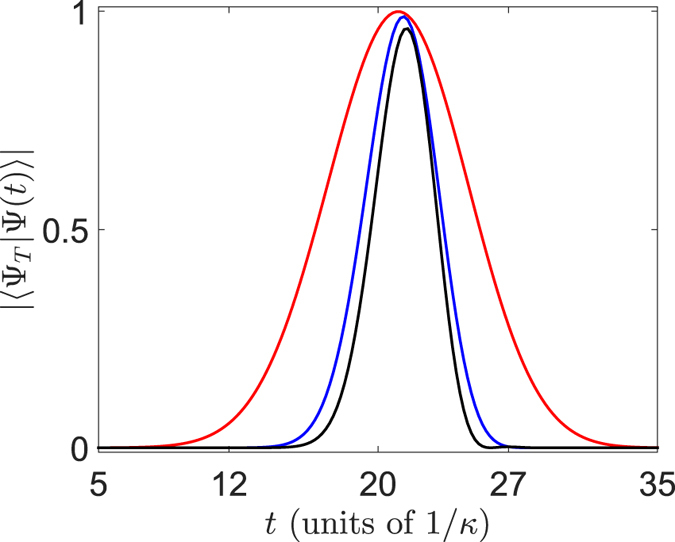
Plots of the fidelity 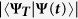 with the parameters *N*_*a*_ = 20, ***N***_***b***_ = 62, *k*_*a*_ = −*k*_*b*_ = *π*/2, in the system Eq. (1) with *N* = 81 and *U* = *v*_*r*_. The red, blue and black lines represent the plots of fidelity 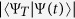 in the condition of 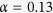, 0.26, and 0.33, respectively. It shows that the fidelity is close 1, as *α* approaches to 0, which accords with the theoretical analysis in the text.

In order to check the above conclusion, numerical simulation is performed. We define the initial and target states as


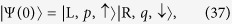



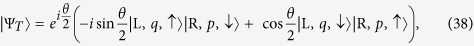


where 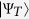 possess the same relative position but the exchanged momentum compared with the state 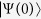 as in Eq. (37). On the other hand, we consider the evolved state 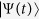 for the initial state being 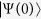 driven by the Hamiltonian (1), and caculate the fidelity 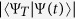 in Fig. 2. It is shown that when the state 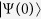 evolves to the same position with 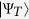, the fidelity 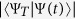 is almost to 1, which is in agreement with our previous theoretical analysis.

**Figure 3:**
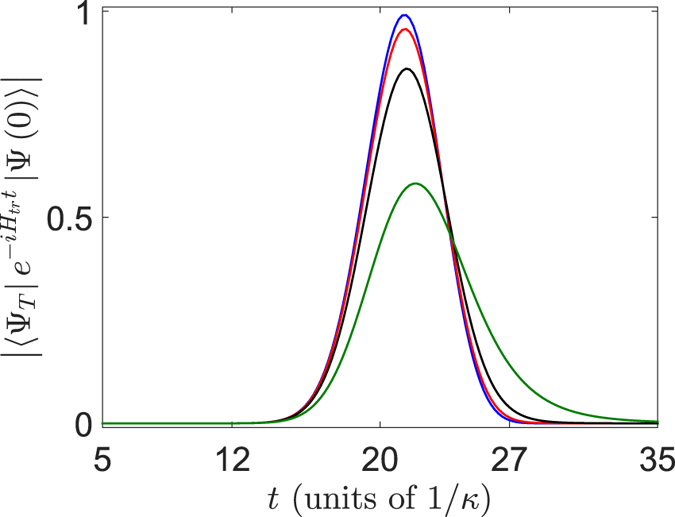
Plots of the fidelity 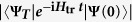 as a function of time *t* with parameters *N*_*a*_ = 20, *N*_*b*_ = 62, *k*_*a*_ = −*k*_*b*_ = *π*/2, in the system Eq. (1) with *N* = 81 and *U* = *v*_*r*_. The blue, red, black and green lines represent the plots of fidelity 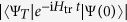 in the condition of 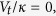 0.0005 0.001 and 0.002, respectively. It indicates that the increase of 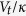 result in decreasing of the maximum of fidelity, which accords with the theoretical analysis in the text.

In ultracold atomic gas experiments, an extra harmonic potential is usually introduced to investigate the interaction between the atoms. To make closer connections with experiment, we will study how an trapping potential can effect on the two-particle collision process. The concerned Hamiltonian can be rewritten as


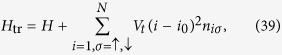


where 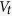 describes an additional (slowly varying) external trapping potential, e.g., a magnetic trap. The presence of the 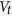 destroy the translation symmetry of the system, therefore one can hardly obtain the analytical result for two-particle collision. Based on this, we perform the numerical simulation to investigate the influence of the external field on the results obtained in a potential free system. In Fig. 3 , we plot the fidelity 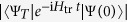 as a function of time *t*, where 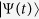 is driven by the Hamiltonian 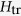. The parameter 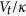 is chosen from 10^−4^ to 10^−3^ for a nearly realistic confinement^15^,^25^,^26^,^27^. It can be shown that the increase of the ratio of 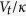 leads to decreasing of the maximum of fidelity. This can be explained as follow: When the strength of the trapping potential 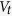 is much smaller than the hopping constant 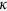, the moving particle will not feel the variation of potential between the adjacent sites, especially at the center of trapping potential. If we consider the collision process at such region, the collision for the two particles only occurs at the neighbour sites due to the short-range interaction between the two particles. Thus, the effect of the confining harmonic potential can be neglected within the collision process.

**Figure 4:**
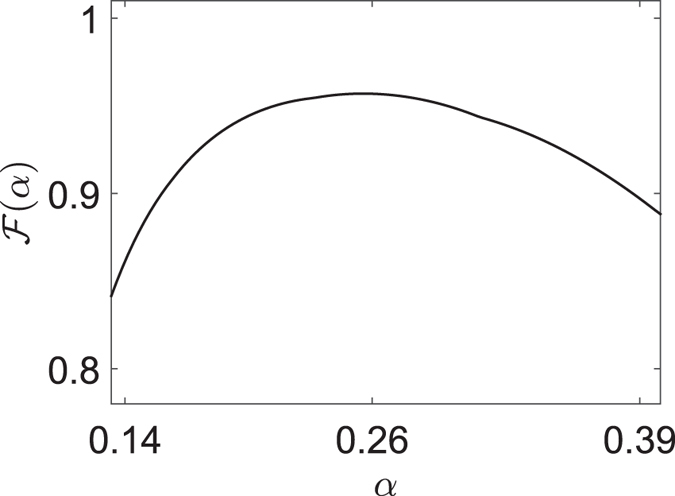
Plot of the function 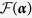 with respect to *α* with parameters *N*_*a*_ = 20, *N*_*b*_ = 62, *k*_*a*_ = −*k*_*b*_ = *π*/2, in the system Eq. (1) with *N* = 81 and *U* = *v*_*r*_. The system is subjected to a weak trapping potential 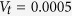 in units of 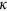. One can see that the maximum of fidelity 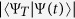 first gets to 0.956 then decrease to 0.88 as the increase of *α*, which can be explained in the text.

On the other hand, the Gaussian wavepacket is often employed to describe the moving particle in the lattice model. The presence of the trapping potential can change the shape and momentum of the moving wavepacket. When the strength of the trapping potential is much smaller than the hopping constant, if the time for the collision process is short enough, the trapping potential can be deemed as a homogeneous field to the involved particles and therefore can not effect on the collision process. For a given weak harmonic trapping potential, the wider of the wavepacket can lead to more time for finishing the collision process, in which the trapping potential can not be neglected. Nevertheless, one can not require the wavepacket to be narrow enough because it can make the two-particle state not to distribute mainly in the invariant subspace of center momentum 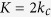, thereby preventing the separation between the spatial and spin part of the two-particle collision. Based on this, the existence of the obtained results regarding the two-particle collision process is a tradeoff between the strength of the harmonic potential and the width of the wavepacket. To check this conclusion, the function 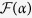 is introduced to characterize the variation of the maximum of fidelity 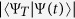 with respect to the width of the wavepacket 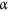, which can be defined as


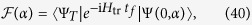


where 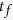 is the time that 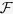 takes to reach the maximum value for a given *α*. In Fig. 4, we plot 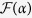 versus *α*, from which one can see that the function 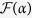 first increase then decrease as the variation of *α*. According to the above analysis, the decrease of width of the wavepacket can bring about two effects: On the one hand, the presence of the trapping potential can be approximately deemed as a homogeneous field due to the slight variation of trapping potential in effective collision process. This can enhance the 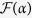. On the other hand, the initial state will not distribute mainly in the invariant subspace 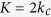. Therefore, the spatial and spin part are not separable within the collision process, which can decrease the 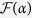. As 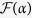 increase, the former effect is dominant. On the contrary, the latter effect is dominant as 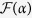 decrease. Moreover, the maximum of 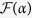 can be seen as a tradeoff between two such effects in this point of view.

Combining both aspects, we can conclude that if the strength of the external trapping potential is much smaller than the hopping constant and the width of the wavepacket is optimum, the results for collision process in our manuscript are still hold.

### Multiple collision

We apply our result to many-body system. Considering the case that the initial state is consisted of many separable local particles with the same group velocity, termed as many-particle wavepacket train (MPWT), our result can be applicable if each collision time is exact known. In this paper, we only demonstrate this by a simple example. We consider the collision of two MPWTs with particle numbers *M* and *N* 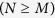. All the distances between two adjacent particles in two trains are identical. The initial state is


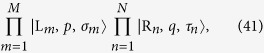


where 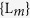 and 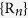 denote the sequences of particles, 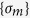 and 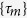 denote the spin configurations in each trains. According to the above analysis, after collisions the final state has the form of


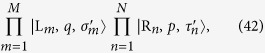


where the spin configurations 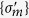 and 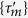 are determined by the S-matrix, which is the time-ordered product of all two-particle S-matrices. During the collision process, the positions of particles in each train are always spaced by equal intervals. This makes it easier to determine the times of each collisions. Then the final state can be written as


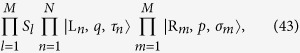


where


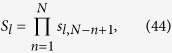


and


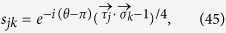


where 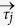
and 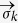 are corresponding Pauli matrices. Applying the formula in Eq. (43) to the case with 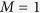, 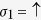, 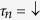, 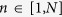, we obtain


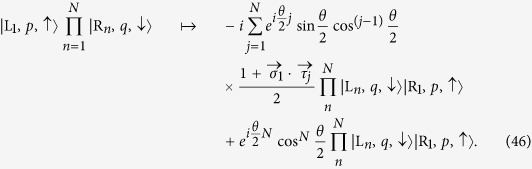


**Figure 5:**
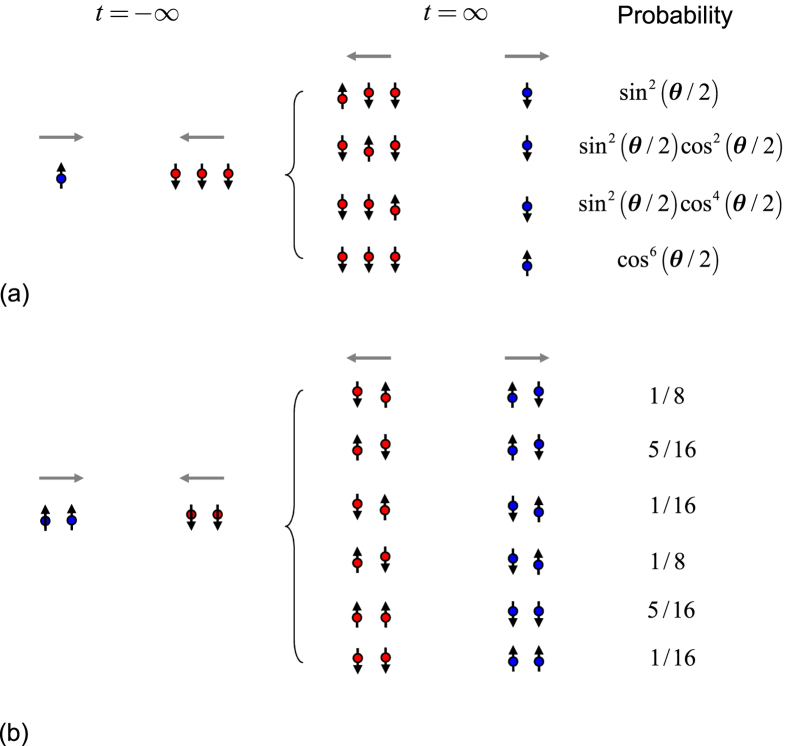
Schematic illustration of the collision between the two MPWTs. (**a**) An incident single fermion comes from the left denoted as blue spin and collides with 3-fermion train, which comes from the right denoted as red spins. It can be seen that the single fermion keep the original momentum, but it entangles with the 3-fermion train at the end of the collision. The amplitudes of the four states are listed. It is shown that the final state is direct product between the states of single fermion and 3-fermion train when 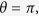 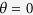 with the corresponding parameter 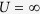, 0, respectively. (**b**) The collision between the two MPWTs come from the opposite direction with particle number 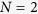. And the probability for the superposition of states is listed with 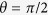.

This conclusion is still true for the case with unequal-spaced 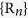. For illustration, we sketch the case with 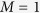, 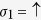, 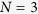, 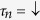, 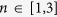 in Fig. 5(a). One can see that the spin part of the final state is the superposition of the combinations of the four spins.

Now we turn to investigate the entanglement between the single fermion and the MPWT with particle number *N*. As is well known, the generation and controllability of entanglement between distant quantum states have been at the heart of quantum information processing. Such as the applications in the emerging technologies of quantum computing and quantum cryptography, as well as to realize quantum teleportation experimentally^28^,^29^. Moreover, quantum entanglement is typically fragile to practical noise. Every external manipulation inevitably induces noise in the system. This suggests a scheme based on the above mentioned collision process for generating the entanglement between a single fermion and the *N*-fermion train without the need for the temporal control and measurement process. We note that although the incident single fermion keep the original momentum, it entangles with the *N*-fermion train after the collision, leading to a deterioration of its purity. To measure the entanglement between the single fermion and the *N*-fermion train, we calculate the reduced density matrix of the single spin


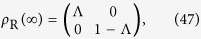


where


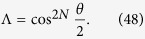


Thus the purity of the single fermion can be expressed as


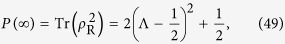


where Tr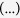 denotes the trace on the single fermion. For the case of 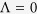, 1, we have 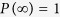, which requires 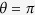, and 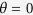, obtained from interaction parameter 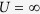, and 0, respectively. It indicates that the single fermion state and *N*-fermion train state are not entangled. In contrast, the purity 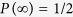 at 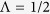 when


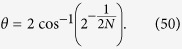


It corresponds to a completely mixed state of the outgoing single fermion, or maximal entanglement between the single fermion state and *N*-fermion train. Together with Eq. (61), we have


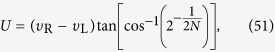


which reduces to 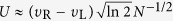 for large *N*. This indicates that for large *N*, one needs to take a small *U* of order 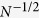 to generate the maximal entanglement between the single fermion state and *N*-fermion train, or result in full decoherence of the single fermion.

In the case of two-train collision, the calculation can still be performed in the similar way. However, it is hard to get analytical result for arbitrary system. Here, we sketch the case with *M* = 2, 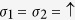, 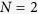, 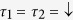, in Fig. 5(b). The probability on each spin configuration is listed as illustration.

## Discussion

Summarizing, we presented an analytical study for two-fermion dynamics in Hubbard model. We find that the scattering matrix of two-fermion collision is separable into two independent parts, operating on spatial and spin degrees of freedom, respectively, when two incident wavepackets have identical shapes. For two fermions with opposite spins, the collision process can create a distant EPR pair due to the resonance between the Hubbard interaction strength and the relative group velocity. The advantage of this scheme is without the need of temporal control and measurement process. Since it is now possible to simulate the Hubbard model via cold fermionic atoms in optical lattice, these results can be realized experimentally.

In conclusion, our finding is of both fundamental and practical interest, as it offers a concrete insight for the fundamental properties of particle paring in the context of the Hubbard model and provide a scheme to realize the distant EPR pair in the experiment.

## Methods

### Symmetry analysis

We analyze three symmetries of the Hamiltonian (1) as following, which is critical for achieving a two-particle solution. The first is particle-number conservation 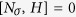, where 
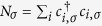
, which ensures that one can solve the eigen problem in the invariant subspace with fixed 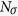, no matter *U* is real or complex. The second is the translational symmetry, 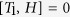, where 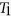 is the shift operator defined as


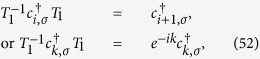


with


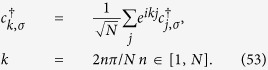


This allows invariant subspace spanned by the eigenvector of operator 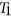. Based on this fact, one can reduce the two-particle problem to a single-particle problem. The final is the SU(2) symmetry, 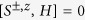 and 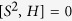, where the spin operators are defined as


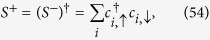



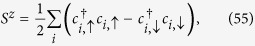


which satisfy the relation 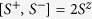.

### Two-particle solutions

We present the detailed caculation for the two-particle solution in each invariant subspace. For the simplicity, we only focus on the solutions in subspaces 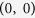 and 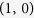, since the one in subspace 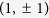 can be obtained directly from that in subspace 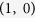 by operator 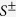. A two-particle state can be written as


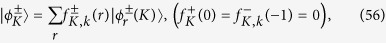


where the wave function 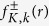 satisfies the Schrödinger equations


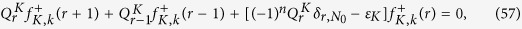


and


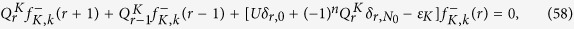


with the eigen energy 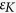 in the invariant subspace indexed by *K*. Here factor 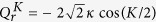 for 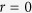 and 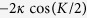 for 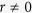, respectively. As pointed in refs 30,31 in previous works, the eigen problem of two-particle matrix can be reduced to the that of single particle. We see that the solution of (58) is equivalent to that of the single-particle 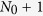-site tight-binding chain system with nearest-neighbour (NN) hopping amplitude 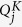, on-site potentials *U* and 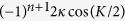 at 0th and 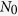
th sites, respectively. The solution of (57) corresponds to the same chain with infinite *U*. In this work, we only concern the scattering solution by the 0th end. In this sense, 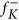 can be obtained from the equivalent Hamiltonian


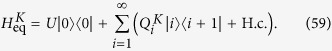


Based on the Bethe ansatz technique, the scattering solution can be expressed as


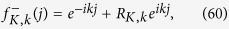


with eigen energy 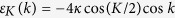, 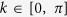. Here the reflection amplitude


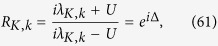


where


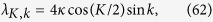



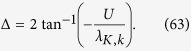


and 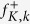 can be obtained from 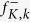 by taking 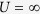. We note that 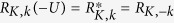, which reveals a dynamic symmetry of the Hubbard model with respect to the sign of *U*.

## Additional Information

**How to cite this article**: Zhang, X. Z. and Song, Z. EPR pairing dynamics in Hubbard model with resonant U. *Sci. Rep.* **6**, 18323; doi: 10.1038/srep18323 (2016).
